# Peptides En Route from Prebiotic to Biotic Catalysis

**DOI:** 10.1021/acs.accounts.4c00137

**Published:** 2024-07-17

**Authors:** Klára Hlouchová

**Affiliations:** † Department of Cell Biology, Faculty of Science, Charles University, Prague 12800, Czech Republic; ‡ Institute of Organic Chemistry and Biochemistry, Czech Academy of Sciences, Prague 16610, Czech Republic

## Abstract

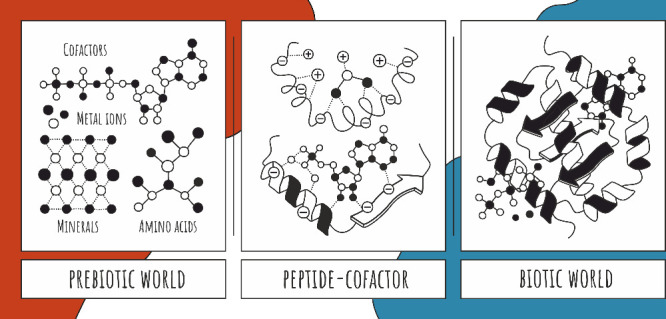

In the quest to understand prebiotic catalysis, different molecular entities, mainly minerals, metal ions, organic cofactors, and ribozymes, have been implied as key players. Of these, inorganic and organic cofactors have gained attention for their ability to catalyze a wide array of reactions central to modern metabolism and frequently participate in these reactions within modern enzymes. Nevertheless, bridging the gap between prebiotic and modern metabolism remains a fundamental question in the origins of life.

In this Account, peptides are investigated as a potential bridge linking prebiotic catalysis by minerals/cofactors to enzymes that dominate modern life’s chemical reactions. Before ribosomal synthesis emerged, peptides of random sequences were plausible on early Earth. This was made possible by different sources of amino acid delivery and synthesis, as well as their condensation under a variety of conditions. Early peptides and proteins probably exhibited distinct compositions, enriched in small aliphatic and acidic residues. An increase in abundance of amino acids with larger side chains and canonical basic groups was most likely dependent on the emergence of their more challenging (bio)synthesis. Pressing questions thus arise: how did this composition influence the early peptide properties, and to what extent could they contribute to early metabolism?

Recent research from our group and colleagues shows that highly acidic peptides/proteins comprising only the presumably “early” amino acids are in fact competent at secondary structure formation and even possess adaptive folding characteristics such as spontaneous refoldability and chaperone independence to achieve soluble structures. Moreover, we showed that highly acidic proteins of presumably “early” composition can still bind RNA by utilizing metal ions as cofactors to bridge carboxylate and phosphoester functional groups. And finally, ancient organic cofactors were shown to be capable of binding to sequences from amino acids considered prebiotically plausible, supporting their folding properties and providing functional groups, which would nominate them as catalytic hubs of great prebiotic relevance.

These findings underscore the biochemical plausibility of an early peptide/protein world devoid of more complex amino acids yet collaborating with other catalytic species. Drawing from the mechanistic properties of protein–cofactor catalysis, it is speculated here that the early peptide/protein–cofactor ensemble could facilitate a similar range of chemical reactions, albeit with lower catalytic rates. This hypothesis invites a systematic experimental test.

Nonetheless, this Account does not exclude other scenarios of prebiotic-to-biotic catalysis or prioritize any specific pathways of prebiotic syntheses. The objective is to examine peptide availability, composition, and functional potential among the various factors involved in the emergence of early life.

## Key References

- TretyachenkoV.; VymetalJ.; NeuwirthovaT.; VondrasekJ.; FujishimaK.; HlouchovaK.Modern and prebiotic amino acids support distinct structural profiles in proteins. Open Biology2022, 12, 22004035728622 10.1098/rsob.220040PMC9213115.^1^*Comparison of random sequence libraries formed from the full and early alphabet shows (i) that they have comparable secondary structure propensities. The early one stands out by (ii) high inherent solubility and (iii) formation of compact structures, independent of chaperones.*
- MakarovM.; Sanchez RochaA. C.; KrystufekR.; CherepashukI.; DzmitrukV.; CharnavetsT.; FaustinoA. M.; LeblM.; FujishimaK.; FriedS. D.; HlouchovaK.Early selection of the amino acid alphabet was adaptively shaped by biophysical constraints of foldability. J. Am. Chem. Soc.2023, 145 ( (9), ), 5320–532936826345 10.1021/jacs.2c12987PMC10017022.^2^*We systematically compared 25-mer random peptide libraries of prebiotic relevance, including some of the most abundant noncanonical amino acids. The canonical early acidic subset of the potential peptide alphabet alternatives stands out by its structure-forming potential*.
- GiacobelliV. G.; FujishimaK.; LepsikM.; TretyachenkoV.; KadavaT.; BednarovaL.; NovakP.; HlouchovaK.; MakarovM.In vitro evolution reveals non-cationic protein – RNA interaction mediated by metal ions. Mol. Biol. Evol.2022, 39, msac03235137196 10.1093/molbev/msac032PMC8892947.^3^*A rRNA-binding domain was engineered to an early composition, lacking any basic and aromatic residues and enriched in acidic residues. The RNA-binding interaction depended on involvement of metal ions, representing a potential early life alternative of this important collaboration*.
- Sanchez RochaA. C.; MakarovM.; NovotnyM.; HlouchovaK.Coenzyme-Protein Interactions since Early Life. eLife2024, 13, RP9417410.7554/eLife.94174PMC1267790041342454.^4^*A PDB-wide analysis of protein–coenzyme interactions uncovered a higher involvement of early amino acids in binding of evolutionary ancient coenzymes. This interaction happens more frequently via protein backbone groups and is more often assisted by metal ions*.

## Introduction

Extant cells depend on hundreds of highly efficient chemical reactions, at any given time of their existence. These processes hinge on the catalytic powers of enzymes, which can accelerate the reaction rates by remarkable factors reaching 10^11^–10^16^ compared to uncatalyzed reactions. Consequently, enzymes play a key role in facilitating the proficiency of today’s biological systems, owing to their selective and efficient mode of action. Catalysts of such nature were absent during the nascent stages of life’s emergence from the prebiotic environment, posing one of the puzzles at the boundary between nonviable and viable eras of Earth’s history. So how did life cross the barrier to cellular life?

The hypotheses regarding the “RNA world” in the origins of life propose that catalytic RNAs could have facilitated early metabolic processes prior to the emergence of proteins which would later assume the primary catalytic roles. Nevertheless, a number of challenges accompany these scenarios, such as RNA synthesis in significant quantities and in the absence of efficient catalysts, and a limited scope of RNA catalytic capabilities (based on our current understanding). Presently, RNA is predominantly recognized for its ability to facilitate peptide bond formation and phosphoryl transfer reactions.^5^ Although the range of reactions may potentially be expanded through interaction with diverse cofactors, contemporary ribozymes often exhibit intramolecular activity and are limited in their efficacy for multiple turnover reactions. Consequently, while ribozymes could have contributed to a certain scope of prebiotic catalysis, they are unlikely candidates for sustaining the majority of early biotic catalytic processes.

In recent years, research in prebiotic chemistry has supported a long-standing hypothesis that a wide array of life’s chemical processes may have been facilitated by metal and mineral catalysts and small organic building blocks.^6,7^ These catalysts were not only abundantly present in the prebiotic environment of early Earth but have also been shown to replicate some reactions of core biochemical pathways experimentally, including parts of the reductive tricarboxylic acid (rTCA) cycle and the Acetyl-CoA pathway, and amino acid synthesis reactions.^6,8^ This collection of reactions could have plausibly initiated complex networks subject to chemical evolution. However, a crucial question remains unanswered. How could such networks transition to biocatalysis as known today, given that the catalytic acceleration rate of metals/minerals is limited to several orders of magnitude (e.g., ref (9)). Could cofactor-binding peptides fill the essential gap?

Approximately half of contemporary enzymes incorporate inorganic, organic, or both types of cofactors.^10^ Several protein scientists have observed that distinct protein folds, particularly ancient ones, can be traced back to shared polypeptide/peptide-long motifs termed “bridging” themes.^11,12^ These motifs suggest a link between present-day biology and its prebiotic origins. Furthermore, the prebiotic abundance of amino acids and their conceivable condensation process render peptides as one of the primary prebiotically plausible molecular entities. Their structural and catalytic potentials, as well as their ability to bind various cofactors, have received limited attention in relevance to prebiotic catalysis and constitute one of the challenges of the contemporary systems chemistry approaches to unraveling the origins of life.

This Account is devoted to exploring the plausible composition, structural characteristics, and functional propensities of early peptides/proteins. Rather than undertaking an exhaustive review of all literature within this domain, the primary objective here is to stimulate additional research focused on bridging the gap between prebiotic chemistry and biochemistry, particularly during the initial ∼500–800 million years of Earth’s history. Emphasis is placed on elucidating the potential catalytic functions that peptides could have performed through concerted interactions with both inorganic and organic cofactors, that would close the gap to the emergence of enzymes as recognized in contemporary biochemistry.

## Proteins-to-Peptides

Although more than 10,000 distinct αβ-folds have been implied possible using our protein alphabet, our biology seems to use a very restricted subset of the protein fold space.^13^ While several different explanations to this paradox may exist, it seems probable that protein evolution has been heavily biased by ancestral relationships, at least for the majority of proteins that we see today. For example, there is a very restricted number of folds (<10) that account for more than 30% of PDB.^14^ At the same time, some of the most ancient domains are among these, such as TIM-barrel, flavodoxin, and ferredoxin-like folds.^14^ Relatively short (approximately 10–40 amino acid long) similar sequence segments were recently detected within such domains, suggesting that an ancestral set of peptides gave rise to numerous seemingly independent domains.^11,12^ Moreover, a lot of these “bridging themes” are associated with the ability to bind cofactors or RNA. It has therefore been proposed that binding to ligands and cofactors could stand at the starting line of different domain emergence and protoenzyme function.^12,15,16^ In further support of this hypothesis, many of the domains that likely predated the Last Universal Common Ancestor (LUCA), such as P-loop NTPases, TIM beta/alpha-barrels, oligonucleotide/oligosaccharide-binding (OB), and Rossmann folds, have been found to harbor prebiotically plausible coenzymes.^17−19^

The occurrence of polypeptides is sometimes associated with the emergence of ribosomal synthesis. Nevertheless, several routes of nonenzymatic peptide synthesis have been proposed and tested, the most effective ones including wet–dry cycles in the presence of ions and condensation of amino acids by minerals. These mechanisms of peptide syntheses are rather nonspecific and can produce nonlinear polymers.^20^ Alternatively, several prebiotically plausible amino acid condensing agents have been described and summarized in depth in a recent review.^20^ Of these, much attention has been devoted to carbonyl sulfide (COS), gaseous compound that is released from volcanoes and deep sea vents. Up to 15-mer peptides have been formed by COS-activated polymerization, the yield reaching 34% of the total amino acid content.^21^ Yields of different amino acid polymerization reactions range from <1% to ∼70%, depending on experimental conditions. Overall, polymerization has been observed significantly more efficient when performed in cycling events, such as in case of the previous yields reported by Greenwald et al. (using continuous addition of amino acids) or wet–dry cycling experiments.^20,21^ In such set-ups, 10–15-mer polymers have been reported, producing chains with a potential to form secondary structures and intermolecular interactions. Although different amino acids have different reactivities during polymerization, the resulting sequences would be also largely affected by the abundance and hence the source of amino acids in the environment of the polymerization.^20^

## Prebiotic Peptide Composition

Given their fundamental importance in life, the prebiotic synthesis of amino acids stands as a central task in prebiotic chemistry. Amino acids, along with their analogs such as hydroxy and dicarboxylic acids, may have accumulated on early Earth from diverse exogenic sources; their presence in interstellar objects suggests the potential for their widespread chemical synthesis throughout the universe.^22,23^ On Earth, amino acids could have been further synthesized from precursor molecules including ammonia, hydrogen cyanide (HCN), and carbonyl compounds via processes such as the Strecker synthesis. Alternatively, transamination and reductive amination of prebiotically plausible α-ketoacid precursors, accessible through prebiotic versions of the reverse tricarboxylic acid (rTCA) cycle, could have occurred using ammonia or hydrazine as nucleophilic nitrogen sources under basic or acidic conditions, respectively.^6^ As argued further below, such chemical networks could have gradually selected for the canonical amino acid alphabet. Collectively, these diverse pathways for amino acid delivery or syntheses suggest their omnipresence in various environments conducive to the origins of life where they could also contribute to simple catalytic or stabilization functions.^24,25^

**Figure 1:**
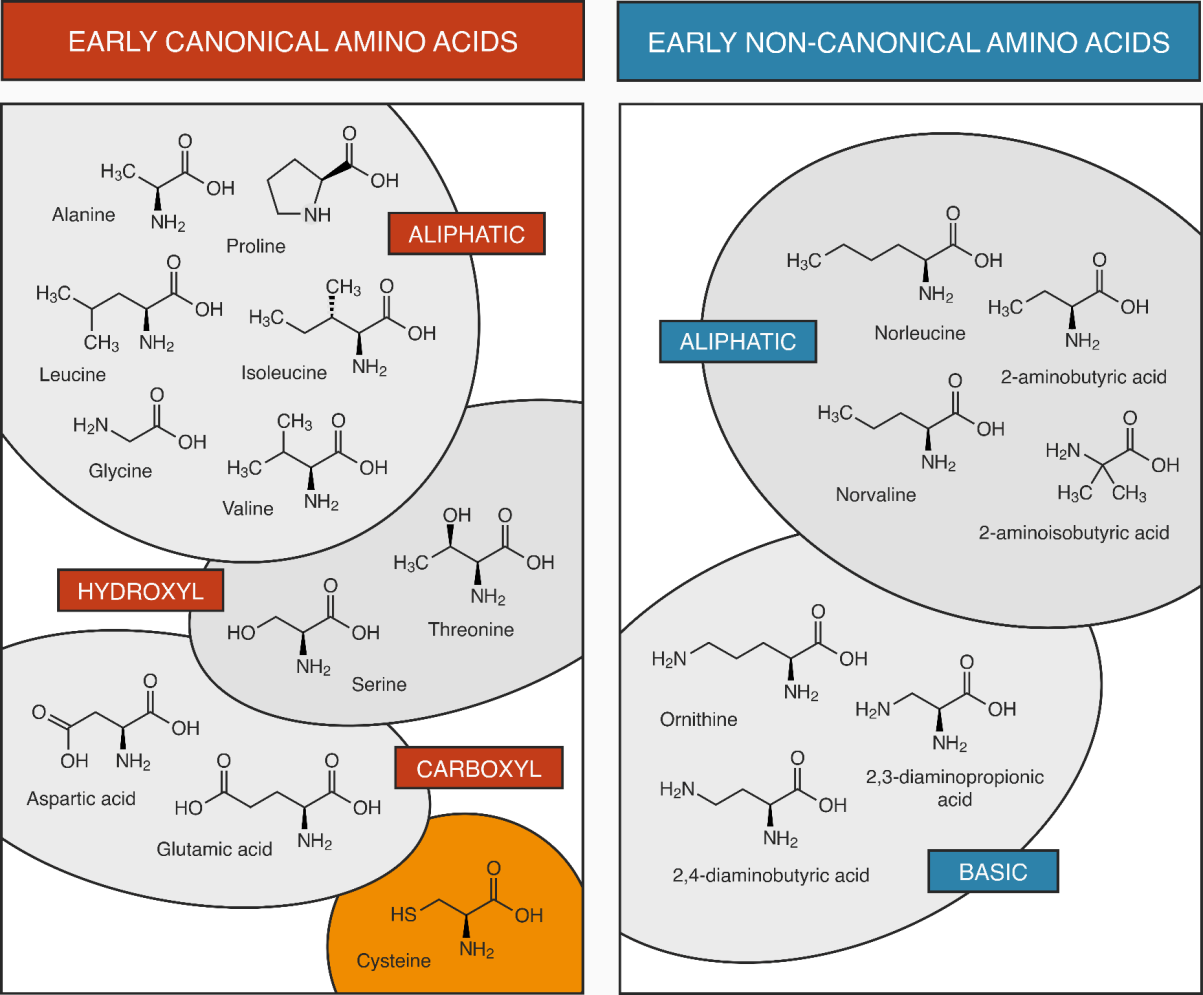
An overview of the α-amino acids detected most abundantly in prebiotic material/experiments;^26,31^ Cys is highlighted in orange as one of the debated candidates.

Nonetheless, distinct sources of amino acids could have imparted different compositional biases to early peptides. Close to 100 different amino acids (mainly α-, β-, and γ-) and their analogs have been detected in meteorites.^26^ Similarly, their syntheses from gases and simple organic compounds also typically yield a number of noncanonical amino acids and hydroxy acids.^20^ At the same time, only about one-half of the canonical amino acids of today’s protein alphabet has been typically detected among these.^22^ This “early” canonical set generally includes the smaller α-amino acids of today’s alphabet bearing (i) aliphatic side chains (Gly, Ala, Leu, Ile, Val, Pro), (ii) hydroxyl groups (Ser, Thr), and (iii) carboxyl groups (Glu, Asp) (Figure 1). Another possible and debated candidate of the “early” alphabet is Cys, as it could remain undetected in many of the mentioned experiments and its prebiotically plausible synthesis has been proposed.^27,28^ Similarly, the other sulfur-containing amino acid Met was detected recently in Miller-Urey experiments that simulated prebiotic atmosphere containing hydrogen sulfide (which was absent in the original experiments) although its synthesis/decomposition rate has been debated.^29,30^ Nevertheless, out of the residues referred to as “early”, about five (Gly, Ala, Asp, Glu, Val) appear systematically in higher quantities than the rest and while additional amino acids appear in some prebiotic sources (such as Phe, Lys, Met or Cys), the early peptide chains would likely be dominated by the “early” amino acids.^31^

Depending on the mechanism and environment of peptide synthesis, various noncanonical amino acids could be incorporated into early peptides. The most abundant noncanonical α-amino acids include α-amino-*n*-butyric acid (ABA), α-aminoisobutyric acid (AIB), norvaline (Nva), and norleucine (Nle).^31^ Analogs to the canonical positively charged amino acids could include their shorter variants (with a smaller number of methylene groups) such as ornithine (Orn), 2,4-diaminobutyric acid (DAB) and 2,3-diaminopropionic acid (DAP) (Figure 1). Such basic side chains would be of limited stability in peptide chains,^32^ but they could provide important moieties e.g. for interaction with cofactors and coacervation.^28,33^ Similar to β- and γ-amino acids, the prebiotically plausible hydroxy acids could form mixed polymers along with the more abundant α-amino acids. These would be less likely to form secondary and tertiary structures but could present oligomers of possible prebiotic relevance. Hydroxy acids could form depsipeptides with amino acids when both ester and amide bonds would be formed in the polymers, e.g. during wet–dry cycle mechanism. Such polymers have been shown to be gradually enriched with more stable amino acids via ester–amide bond exchange and thus represent yet another possible route from prebiotic constituents to peptides.^34^

The process by which canonical amino acids were selected from the primordial pool, and its possible supplementation by other amino acids that would become more abundant through later (bio)synthesis, remains uncertain. Factors such as (un)reactivity and stability within peptide products seem possible. One conceivable scenario suggests that early chemical evolution toward peptide structural propensity influenced the composition of early peptides and favored selection of the canonical amino acids.^2^ Another intriguing hypothesis involves interaction of amino acids with nucleotides, potentially directly transferring to the genetic coding system. In connection with the synthesis of amino acids from α-ketoacid precursors as described earlier, it has been suggested that the interaction of these precursors with dinucleotides could not only catalyze the reaction but also elucidate the relationship between individual amino acids and their codons.^35^ It has been hypothesized (yet not experimentally supported) that 14 of the canonical amino acids could be synthesized in this manner and traced back to the dinucleotide codon, excluding Trp, Tyr, Phe, Lys, His, and Met. The most straightforward synthesis proposed would involve the direct reductive amination of an α-ketoacid, potentially catalyzed by the exocyclic amino group of G in the first position and the exocyclic amino groups of G, C, or A.^35^ Intriguingly, if true, this process would yield (and encode) the amino acids Gly, Ala, Asp, and Glu, which resemble those amino acids most frequently found in other conceivable prebiotic sources.

It is probable that the various sources of amino acids and peptides described above, along with potential additional sources, could have been operative in parallel on early Earth in diverse environments such as hydrothermal vents or surface ponds. Further systematic experiments may lead us to connections between specific amino acids and plausible modes of peptide syntheses at the different sites. Nevertheless, our current knowledge suggests that peptides composed of simpler amino acids, particularly enriched in acidic residues, were likely most prevalent. Furthermore, it is probable that these amino acids were initially accompanied by noncanonical small residues that were more abundant via prebiotic synthesis. Some of these residues could have participated in primitive translation by early “generalist” versions of aminoacyl tRNA synthetases (AARSs), as proposed and partially documented by their reactivity with AARSs e.g. for norvaline, ornithine and alpha-aminobutyric acid.^36,37^ A recent analysis suggests that “specialist” AARSs diverged from this generalist pool to incorporate the larger, presumably “late” side chains, as these became more abundant and selected into the genetic coding.^37^ Due to the diminished stability of basic residues with shorter side chains (such as DAB and ornithine) within polymers, it is improbable that these residues were highly abundant in early peptides or proteins.^32^

Independent of the amino acid composition, the emergence of peptides/proteins is surrounded by the enigma of their single chirality. It remains unresolved whether this chirality was established through abiotic processes at the monomer level (e.g., by the effect of magnetized surfaces), or if it was selected during the prebiotic transition from heterochiral ligation to homochiral enrichment.^38,39^ This question continues to pose a significant challenge in the field.

## Early Peptide/Protein Structural Propensities

In 1975, Brack and Orgel pointed out that the most frequently occurring prebiotic amino acids would likely be Gly, Ala, Asp, and Glu, with the potential addition of Ser and Thr.^40^ Unless specific amino acids would be selected from the prebiotic pool, they argued that products of early prebiotic condensation would favor β-sheet structures, based on the known tendencies of polypeptides formed from repeating units such as Glu-Ala, Gly-Ala, and Gly-Ser.^40^ This early assertion finds support in the proposal that β-sheet structures may have emerged earlier than α -helices in biological life, as inferred from the analysis of proteins accreted from the center to the surface of the ribosome.^41^

In recent years, we have devoted substantial effort to elucidating the structural implications of various plausible compositions of early peptides and proteins through systematic screening of random sequences. Although some exemplary mechanisms of primitive templating of amino acid condensation have been proposed (e.g., ref (42)), the widespread peptide formation likely relied on their random incorporation prior to the establishment of advanced ribosomal synthesis. We previously demonstrated that even random sequences comprising the canonical protein alphabet (i.e., the 20 proteinogenic amino acids) exhibit similar secondary structure content as biological proteins (within 5% difference overall), featuring similar ratios of α-helices and β-sheet. We further concluded that this is due to inherent secondary structure propensities of this set, unlike for some of its alternative subsets, including e.g. homologous noncanonical amino acids, as described further below.^2,43^

**Figure 2:**
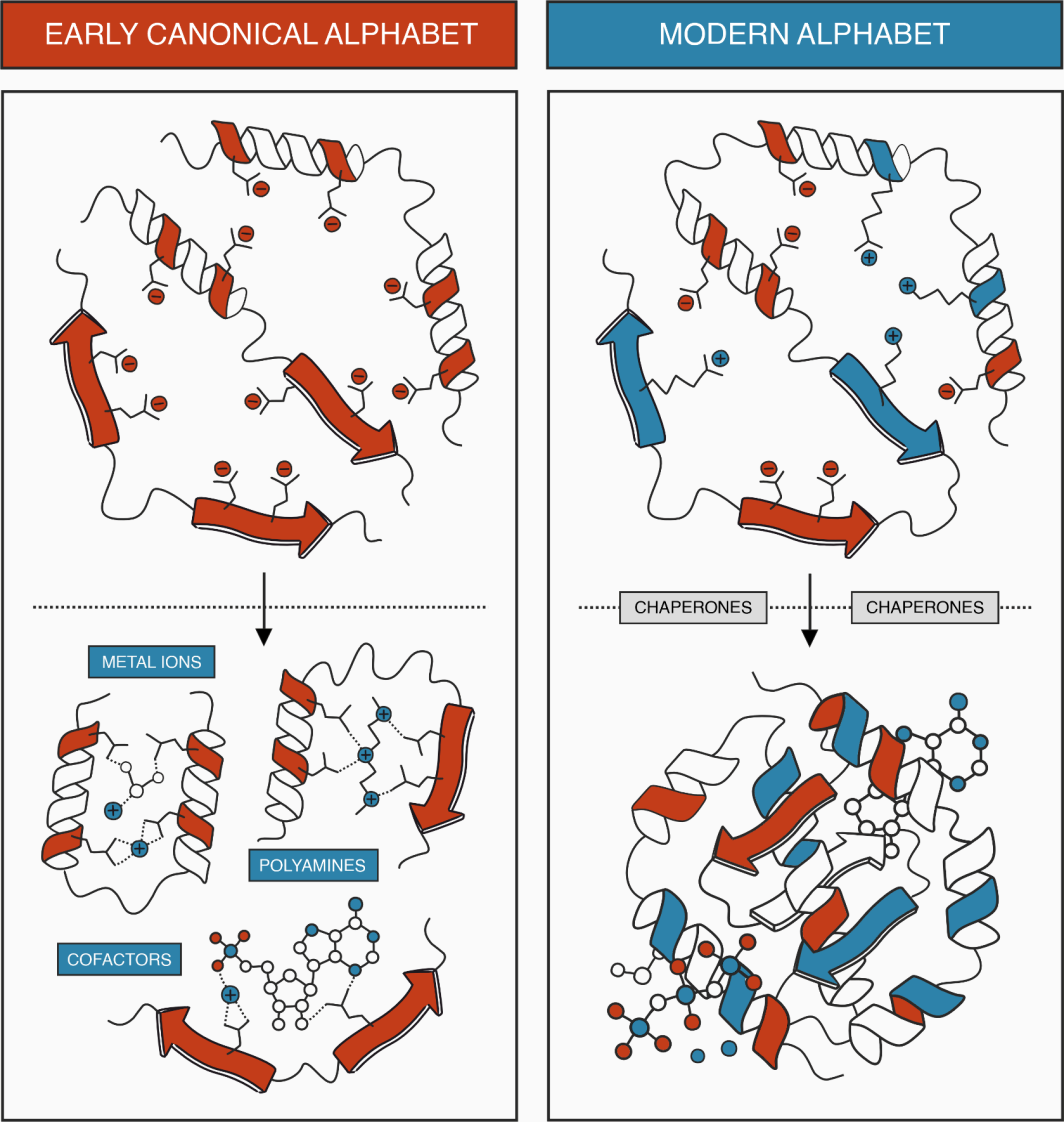
A model scenario of how structure formation is yielded for the presumed “early” acidic peptides (left) and modern proteins (right).

Employing domain-size random sequence libraries composed of the 10 reduced “early” amino acids (Ala, Asp, Glu, Gly, Ile, Leu, Pro, Ser, Thr, Val), we compared the structural propensities of these residues with those of contemporary amino acids.^1^ While a moderate enrichment of α-helices was noted in the contemporary alphabet, the reduced “early” amino acid library displayed comparable overall secondary structure and compaction propensity. Furthermore, the reduced “early” alphabet library exhibited significantly greater solubility, contrasting with the dependence of contemporary alphabet solubility on chaperones (Figure 2).^1^

The specific reduced “early” alphabet utilized in our study was based on the most recent meta-analysis.^22^ While subtle discrepancies arise in alternative studies of prebiotically most plausible amino acids, most agree on an acidic alphabet enriched in small and primarily aliphatic amino acids. Most scenarios lack aromatic residues conducive to folding, as well as positively charged residues. It is thought provoking that such amino acids yield similar structure-forming propensities as the canonical set of 20, raising the possibility that these may represent inherent properties of any analogous set of α-amino acids. However, subsequent studies have challenged this assumption.

Using alternative formulations of the “early” random library, which incorporated noncanonical basic residues or variations of small noncanonical aliphatic residues, our findings suggest that the choice of presumed “early” amino acids to comprise today’s protein alphabet would likely not be arbitrary. Specifically, our investigation revealed that the presumed “early” acidic subset of the contemporary alphabet exhibits a distinctive capacity to form α-helical and β-sheet motifs, whereas alternative compositions decreased this property, at least at the level of 25-mer peptides examined in our study.^2^

Recent work by Despotovic et al. proposed that prebiotically plausible polyamines, and potentially metal ions, could induce protein folding in the absence of basic residues.^44^ Concurrently, the Hecht group demonstrated that binding of metal ions is a surprisingly frequent occurrence in unevolved sequences.^45^ While our comparative study of contemporary versus the presumed “early” alphabet libraries was conducted in a cell-like *in vitro* expression environment where these factors may have contributed to the structural propensities of the early alphabet,^1^ it is noteworthy that these chemical entities were largely excluded in our comparison of 25-mer libraries, as these were synthesized via solid-phase peptide synthesis.^2^ Consequently, while folding of “early” acidic sequences may be induced by polyanions and metal ions, the intrinsic secondary structure propensity and solubility of such peptide compositions remain even in their absence (Figure 2).

It is important to stress here the difference between secondary structure propensity and the ability to form tertiary structure, which would be a major obstacle in bridging the gap toward protein-like enzymes as we understand them. For peptides with secondary structure potential, this may be achieved partly by their self-assembly. For longer sequences, binding to inorganic and organic cofactors may partly assist in building compact tertiary structures as implied above, even in the absence of hydrophobic core-forming residues.

## Binding to Inorganic Cofactors and Coenzymes

Amidst the evolving diversity of amino acids, the question persists regarding the stage at which peptides “acquired the ability” to interact with cofactors and leverage these interactions toward improved catalytic activities. Could this have occurred prior to templated ribosomal synthesis of peptides and potentially with a reduced amino acid alphabet?

It has long been hypothesized that even short peptides of variable composition would have the capacity to bind biologically important cations and anions. Polymers of 3–4 amino acids, referred to as “nests”, have been proposed to have the propensity to chelate these ions through the main chain amide carbonyl, aided by terminal carboxyl and amine groups, based on similar motifs observed within protein structures.^46^ In the absence of regular structural motifs, particularly α-helices, the peptides would be more exposed to solvent environments. The seminal analysis by Milner-White and Russell suggested that irrespective of the side chain moieties, such peptides would exhibit the capacity to bind various metal ions, phosphate, and even iron–sulfur centers.^46^ While not all such configurations are commonly found in modern proteins, it has been argued that remnants of these “nests” persist in ancient domains or motifs, such as in phosphate-binding P-loops. Notably, recent research has experimentally demonstrated a recreation of a phosphate-binding polypeptide from prebiotically available amino acids, while preserving the basic side chains through Arg-Orn mutations.^33^

As mentioned earlier, regardless of the source of amino acids and peptides, it is anticipated that the earliest peptides were likely enriched in acidic residues. For instance, the Asp-rich Mg^2+^ binding motif DxDGD, which encompasses a nest-like arrangement, persists in structures such as RNA polymerases.^47^ It has been proposed that peptides rich in Asp/Glu could shield RNA from degradation in the presence of high Mg^2+^ concentrations, and potentially contribute to early ribozyme function through acid/base chemistry or stabilization of substrate transition states.^48^ Notably, the prebiotically abundant Mg^2+^ and Ca^2+^ ions predominantly coordinate with early amino acids Asp/Glu in contemporary metalloenzymes, whereas Cu^2+^ and Zn^2+^ ions, considered scarce during the Hadean–Archaean period, are coordinated primarily by apparently later-evolved residues such as His and Cys.^28^ Nevertheless, it is important to note that until establishment of early ribosomal synthesis, amino acids containing two carboxylate groups would be prone to forming branched polymers.

Magnesium ions play a pivotal role in RNA structure formation and have been found to be enriched in the ancient core of the ribosome.^49^ Through a reverse engineering approach utilizing a ribosomal protein L11 RNA-binding domain, we observed that metal ions possess the capability to facilitate interactions between RNA and compositionally reduced proteins, lacking e.g. aromatic and positively charged residues. Specifically, by bridging the acidic Asp/Glu residues and the negatively charged phosphate groups, a variant of the L11 RNA-binding domain composed of the presumed “early” amino acids retains its RNA-binding affinity in the presence of Mg^2+^ ions.^3^ Such interactions may represent potential alternatives to RNA-protein interactions prior to the prevalence of basic amino acids that currently dominate them.^28,50^

The early coexistence and collaboration of nucleotides and amino acids are also evidenced by organic cofactors, i.e. coenzymes, many of which incorporate both of these molecular components.^51^ While not as prevalent as inorganic cofactors, the fundamental units of numerous coenzymes have been identified in experiments simulating prebiotic conditions (although typically not under mutually compatible conditions).^28,52^ These molecules possess the ability to catalyze a wide array of metabolic reactions, some even in the absence of enzymes, potentially associated with micelles/vesicles.^9,53^ Alongside metal ions and minerals, coenzymes are likely part of the earliest catalytic cohort and simultaneously broaden the range of potential catalytic actions (Table 1).^10^ In extant enzymes, they are found across all E.C. classes, underscoring their lasting significance. It is tempting to hypothesize that their interaction with peptides and small proteins could represent the most recent step between prebiotic and biotic catalytic efficiencies.

**Table 1:**
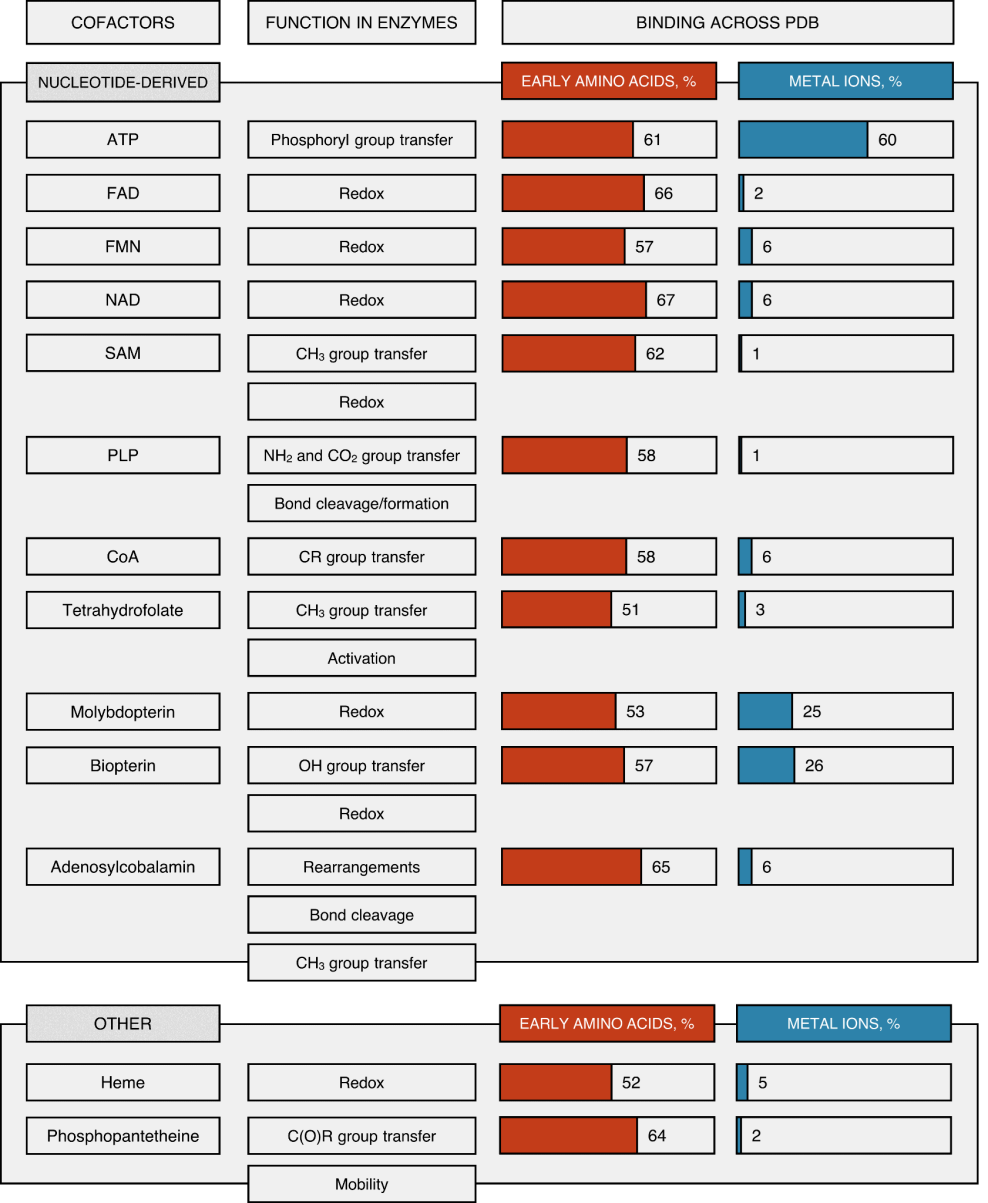
A List of Prebiotically Plausible Organic Cofactors (Divided to Nucleotide-Derived and Other), Their Possible Functions and Binding Properties in Extant Proteins^4,10^^a^

a The percentage of the presumed “early” amino acids average involvement in binding to the cofactors is listed (the average overall content of “early” amino acids across PDB is 67%). For comparison with the presumed later coenzymes, refer to ref (4).

Several recent perspectives have explored Harold White’s proposition that coenzymes, conceived as products of the RNA world by the author, initially formed the earliest catalytic sites of ribozymes and were subsequently transferred to protein enzymes.^18,52,54^ Although coenzymes have been shown to interact with several ribozymes, a definitive causal connection to their requirement for nurturing by ribozymes before engaging with polypeptides appears to be lacking. Coenzymes are typically abundant in ancient proteins, and numerous short and simple coenzyme-binding motifs, presumed to precede enzyme domains, have been identified.^12,15,55,56^ Analogous to the mechanisms of metal ion binding “nests”, it has been suggested that the simplest coenzyme-binding motifs primarily involve backbone amide interactions.^55^

We have recently performed a systematic search throughout the PDB database for all the protein-coenzyme binding events in today’s proteins and differentiated coenzyme classes based on their prebiotic plausibility.^4^ All the analyzed coenzymes bind more preferentially via the presumed “late” amino acids (based on the 67% “early” to 33% “late” amino acid average occurrence in proteins). Nevertheless, the ancient coenzymes, that include many of the nucleotide-based coenzymes but are not restricted to them (Table 1), are more often bound by early amino acids than younger coenzymes.^4^ Indeed, such interactions rely more frequently on the protein backbone amide groups and also involve more metal ions. We detected several examples of ancient coenzyme (such as ATP and NAD) binding which was supported by early amino acids only.^4^

Altogether, these results suggest the plausibility of peptide-coenzyme cooperation prior to involvement of the late amino acids. Nevertheless, further experimental data is needed to quantify such interactions and test whether prebiotically plausible peptides may have the capacity to serve as catalytic intermediaries between minerals, metal ions, and coenzymes on one side and modern enzymes on the other.

## Could Early Peptides Take Part in Prebiotic Catalysis?

We have learnt that peptides and proteins composed of the constrained set of the presumed “early” amino acids have the capacity to form regular motifs and compact structures, respectively, and possibly interact with a variety of organic and inorganic cofactors. The arising question regards the scope and efficiency of catalytic activities that such a molecular repertoire could serve in early metabolism. While this question ultimately requires dedicated experimental investigation, we can cautiously speculate on potential scenarios based on the compiled data.

In today’s proteins, about ten amino acids (specifically Arg, Asp, Cys, Glu, His, Lys, Ser, Thr, Trp, and Tyr) participate directly in catalysis most frequently, enriched by the backbone amide and N’ and C’ termini groups.^57^ Four of these (Asp, Glu, Ser, Thr) are considered among the presumed “early”. Histidine stands out as a residue that is found in the active sites of all enzyme E.C. classes and has the highest catalytic propensity. The second place is occupied by Cysteine that is most prevalent in oxidoreductases, transferases, and isomerases.^57^ Both of these amino acids are considered “late”, although Cys is one of the highly debated ones, as pointed out above, and regarded as crucial for the Fe–S dependent metabolism.^58^ His has been implied as a possible product of very early biological evolution, starting from the same precursors as purine synthesis.^59^ Nevertheless, all the ten residues have been reported to be capable of the majority of the amino acid catalytic roles (activation, steric roles, stabilization, proton/electron/hydrogen shuttling, and covalent catalysis) to some extent.^57^

The most frequent role of amino acids in catalysis involves electrostatic stabilization or activation (mainly by affecting p*K*_a_ and redox potential) of reaction intermediates.^57^ In early polypeptides/peptides, this can be provided by the early Asp, Glu, Ser, and Thr or the backbone amide/carbonyl groups. While Arg, His, and Lys would be probably deficient for this role in early polypeptides/peptides, their functionality could be substituted by metal ions, Asp/Glu or potentially the N’ amine. Nevertheless, this inference is highly speculative and extensive experimental efforts will be required to test the catalytic scope and efficiency under such compositional changes. Even small peptides or their assemblies have been reported to possess catalytic potential.^7,60−63^ Ser-His dipeptide is one of the most debated ones, with activity reminiscent of serine protease hydrolysis. The role of His is in polarization of the nucleophilic Ser, allowing it to perform the nucleophilic attack on the substrate.^7^

In today’s enzymes, some of the catalytic roles are dominated by coenzymes. These include mainly shuttling of protons (although that can also be provided by amino acid residues), hydrides and both single electron and electron pairs.^10^ Coenzymes also stand out in their capacity to form covalent intermediates. A majority of oxidoreductase reactions and many of the transferase reactions rely on the irreplaceable chemistry of coenzymes while many of these coenzymes belong to the prebiotically plausible ones (such as CoA, PLP, FAD, NAD, FMN).^10^ It is therefore probable that these activities would be plausible prebiotically, although with worse efficiencies.

In contemporary catalysis, amino acids play a crucial role in creating highly specific nonpolar sites, facilitating efficient and specific reactions through precise steric arrangements and subtle effects of local environments. The absence of aromatic amino acids, which significantly contribute to the formation of hydrophobic cores among other functions, could present challenges in achieving such specificity. One possible path toward compact arrangements is by intermolecular assemblies or amyloids, accessible to peptides of prebiotically plausible lengths (approximately >5-mers).^21^ Moreover, we recently demonstrated that enzymatic phosphotransferase activity, reliant on nonpolar environments, can be maintained even in the complete absence of aromatic residues, albeit with a notable decrease in efficiency by 2–3 orders of magnitude.^64^ Notably, protein compaction was observed only after substrate binding in this study. Prior to evolutionary optimization and sequence fixation through templated synthesis, early peptide/protein-coenzyme hubs would face similar challenges. At the same time, they could likely present significant advantages e.g. in stabilization and activation of the reaction intermediates, and in creating a compartmentalized environment of the reactions, when compared with the cofactors alone.

In summary, it is plausible to assume that a comparable range of chemical repertoire could be achieved by early peptides/proteins with an increased involvement of both inorganic and organic cofactors. Nevertheless, this hypothesis calls for large experimental efforts to determine the feasibility of such catalytic hubs and their potential range of catalytic rate enhancements.

## Conclusions and Future Outlooks

Previous investigations into the origins of life have amassed extensive knowledge regarding the prebiotically plausible synthesis and reaction scopes of various molecular species crucial to contemporary life or representing potential alternatives during its emergence. Despite this wealth of information, how life emerged from the prebiotic Earth remains elusive even decades later. Recent discourse advocates for a shift in strategy. The dichotomy between the “RNA world” and “metabolism-first” paradigms is fading, with a systems chemistry approach emerging as the next frontier in addressing one of life’s greatest mysteries.^65,66^

Minerals, metal ions, organic cofactors, and ribozymes are usually considered potential members of the early stage of catalysts, each with different catalytic scopes and properties. While considered prebiotically plausible, peptides are usually not shortlisted, due to uncertainties regarding their potential involvement, limited experimental data, and their differentiation from modern proteins. Contemporary enzymes are characterized by geometrically specific, nonpolar active sites that confer high specificity and efficiency. On the other hand, short peptides of random composition (that would be favored before ribosomal synthesis) would unlikely confer such properties. Nevertheless, peptides and their analogs (such as depsipeptides) have been suggested as constituents of early membranes or drivers of coacervation during life’s emergence.^20,67^ They have been observed to interact with RNA and provide structural scaffolding in the ribosome.^49^ Although peptides are acknowledged as potential catalysts, their catalytic potential within the prebiotic environment, in cooperation with other catalytic species, remains significantly understudied.

This Account overviews key literature on probable composition of early peptides and summarizes studies concerned with its implications. The convergence of previous studies suggests that early peptides/proteins were predominantly composed of small, mainly aliphatic and acidic amino acids, potentially accompanied by some noncanonical residues with similar properties. Over the past ∼5 years, research by our group and others has demonstrated that such peptides and proteins possess the capability to form secondary and compact structures, as well as interact with RNA and organic cofactors of prebiotic origin. These interactions are facilitated by metal ions, which inherently exhibit an affinity for such peptides.

It is important to note that these observations do not intend to assert exclusivity or argue against the plausibility of other prebiotic catalysts. On the contrary, peptides may offer additional functionalities to the already recognized prebiotic repertoire. Significantly, they may serve as a direct bridge to enzymes, the primary catalysts of contemporary life, if they enhance the catalytic efficiency of organic and inorganic cofactors alone. Numerous ancient protein domains have been traced back to simple peptide “bridging themes” or “vocabulary”, often possessing cofactor-binding capabilities. These themes may directly relate to peptides of prebiotic origin, as proposed in previous literature.^11,12^ The next step is to conduct systematic experimental tests to validate these hypotheses.
